# Genetics and genomics etiology of nonsyndromic orofacial clefts

**DOI:** 10.1002/mgg3.272

**Published:** 2017-01-17

**Authors:** Wasiu L. Adeyemo, Azeez Butali

**Affiliations:** ^1^ Department of Oral and Maxillofacial SurgeryCollege of MedicineUniversity of LagosSurulereNigeria; ^2^ Department of Oral Pathology, Radiology and MedicineCollege of DentistryUniversity of IowaIowa CityIowa; ^3^ Iowa Institute of Oral Health ResearchCollege of DentistryUniversity of IowaIowa CityIowa

## Abstract

Orofacial clefts (OFC) are complex birth defects. Studies using contemporary genomic techniques, bioinformatics, and statistical analyses have led to appreciable advances in identifying the causes of syndromic forms of clefts. This commentary gives an overview of the important cleft gene discoveries found using various genomic methods and tools.

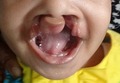

## Introduction

Orofacial clefts (OFC) are complex birth defects. Seventy percent of all clefts are classified as nonsyndromic, where no recognizable structural defects other than cleft are seen. The remaining 30% are syndromic, where a cleft presents with a consistently defined structural anomaly; these are usually Mendelian in nature. Studies using contemporary genomic techniques, bioinformatics, and statistical analyses have led to appreciable advances in identifying the causes of syndromic forms of clefts. Meanwhile, only modest progress has been recorded for nonsyndromic clefts. This commentary gives an overview of the important cleft gene discoveries found using various genomic methods and tools (Table 1). At the end we discuss the discoveries' value for genetic counseling and as foundations for future research.

**Table 1:** A list of some important candidate genes and loci identified for nonsyndromic orofacial clefts and platforms for identification over time

Candidate genes and genomic loci^a^	Technology and methodology	Timeline
*IRF6*	Linkage, Association, Targeted sequencing, GWAS, Exome sequencing, Copy number variation	A, B
*FOXE1*	Linkage, Association, Targeted sequencing, GWAS	A, B
*MSX1*	Targeted sequencing	A
*BMP4*	Targeted sequencing	A
*FGFR1*	Targeted sequencing	A
*FGFR2*	Targeted sequencing	A
*CRISPLD2*	Linkage and Association	A
*SUMO1*	Mutation screen	A
*TFGβ*	Association	A
*MAFB*	GWAS	B
*PAX7*	GWAS	B
*VAX1*	GWAS	B
*ARHGAP29*	GWAS, Mutation Screen	B
Chr8q.24	GWAS	B
Chr16p13.3	GWAS	B
*VAX1*,	GWAS	B
*NOG*	GWAS	B
*GRHL3*	Linkage, Exome sequencing	A, C
*CDH1*	Exome sequencing, Targeted sequencing	B
*MGAM*	Copy number variation	B
*ADAM3A*	Copy number variation	C
*ADAM5A*	Copy number variation	C

A = pre‐GWAS era, B = GWAS era, C = post‐GWAS era.

a This is not an exhaustive list but a representation of some important genes discovered using various statistical approaches and technology in the pre‐GWAS, GWAS, and post‐GWAS era.

## Pre‐Genome Wide Association (GWAS) Era

Our ability to identify candidate genes and risk loci for OFC has changed as genomic technologies have changed. Since the early 1990s, linkage studies were used to identify risk loci in large affected families (Pulst 1999). Linkage was used to map the locus for van Der Woude syndrome (VWS; Murray et al. 1990), which is the most common syndromic cleft, accounting for 2% of all OFCs. It is characterized by OFC and bilateral lower lip pits that are highly penetrant in affected families and inherited in an autosomal dominant fashion (Lam et al. 2010). In 2002, mutations in the *IRF6* gene, which accounts for 75% of VWS (OMIM: 119300) and popliteal pterygium syndrome (PPS; OMIM: 119500), were first reported (Kondo et al. 2002). Resequencing of *IRF6* gene in nonsyndromic OFC (a phenocopy of VWS) identified a missense mutation in *IRF6* p.Val247Iso that was more frequent in cases than controls and significantly overtransmitted in affected families using the family based approach (Zucchero et al. 2004). These findings suggest that common polymorphisms in *IRF6* are associated with nonsyndromic clefts and rare variants in this gene are associated with VWS and PPS. To date, over 350 *IRF6* mutations have been reported for VWS and PPS (De Lima et al. 2009; Leslie et al., 2013; Butali et al. 2014). Nonetheless, these mutations only explain 70% of VWS cases. In the remaining 30% of cases, different risk loci across the genome may be responsible. Linkage was also used to map the second locus for VWS in a large Finnish family confirming the contribution of additional risk loci for VWS (Koillinen et al., 2001).

The contribution of variations in the regulatory region in the genome for syndromic and nonsyndromic clefting has been established. Fakhouri et al. (2014) found a 350dupA mutation in a VWS family which abrogated the binding of p63 and E47 transcription factors to cis‐overlapping motifs. This mutation was shown to be functional since it significantly disrupted a conserved *IRF6* enhancer element (MCS9.7) activity. Prior to this, Rahimov et al. (2008) reported an association between a single‐nucleotide polymorphism (SNP) and nonsyndromic clefts in an *AP2* alpha binding enhancer near the *IRF6* gene. They then tested the functional role of this enhancer in mice and reported that it recapitulates the *IRF6* expression in the craniofacial region (Rahimov et al. 2008).

Fox‐head protein 1 (*FOXE1*) is also a cleft candidate that was discovered through genome‐wide linkage scans and family‐based association studies (Marazita et al. 2004; Moreno et al. 2009). Other candidate genes have been identified from animal studies and from their role in syndromic forms of clefts. These include *MSX1* and *BMP4* (Satokata and Maas 1994; Jezewski et al. 2003; Suzuki et al. 2004, 2009; Butali et al. 2011). Many more candidate genes have been reported to be associated with nonsyndromic clefts using multiple complementary methods in the pre‐GWAS era, including *FGFR1*,* FGFR2* (Riley and Murray 2007), *CRISPLD2* (Chiquet et al. 2007), *SUMO1* (Alkuraya et al. 2006) and *TFGβ* (Ardinger et al.^1989^) to mention a few.

## GWAS Era

As with other complex traits, genome‐wide association studies (GWAS) have been used to identify new risk loci for clefting. First, GWAS confirmed two genes previously identified as likely to be responsible for clefting. *IRF6* was confirmed as having a role in nonsyndromic OFCs when significantly associated loci were reported (Birnbaum et al. 2009; Grant et al. 2009; Beaty et al. 2010; Mangold et al. 2010; Ludwig et al. 2012; Leslie et al. 2016); these studies increased the evidence pointing to *IRF6* as a clefting gene. Furthermore, GWAS studies validated the assumption that common variant in *IRF6* are associated with nonsyndromic clefts. In addition, a GWAS recently confirmed significant association for SNPs in *FOXE1*, thus providing evidence that this gene is involved in nonsyndromic OFC etiology (Ludwig et al. 2014). Validation of the role of these two previously identified candidate genes by an unbiased genomic method such as GWAS is a critical step toward genetic counseling for families.

Besides validating previously identified gene candidates, GWAS have identified significant signals in *MAFB* and near‐significant loci in *PAX7* and *VAX1,* which have been replicated in multiple populations (Beaty et al. 2010; Butali et al. 2013a; Mangold et al. 2010; Nasser et al., 2012). In 2012, *ARHGAP29* was also reported as a cleft candidate gene following resequencing around *ABCA4*, a GWAS‐candidate gene (Beaty et al. 2010; Leslie et al. 2012).

GWAS have also identified several susceptibility loci. A meta‐analysis of GWAS studies replicated previous findings and identified six new candidate loci (Ludwig et al. 2012). The Chr8q.24 locus has been reported to be significantly associated with nonsyndromic OFC in GWAS and replication studies using samples from populations of European descent (Birnbaum et al. 2009; Grant et al. 2009; Beaty et al. 2010; Mangold et al. 2010; Ludwig et al. 2012) and in Africans (Gowans et al., 2016). A GWAS in the Chinese population identified a new susceptibility locus for nonsyndromic OFC at the Chr16p13.3 locus between the *CREBBP* and *ADCY9* genes (Sun et al. 2015). This GWAS also confirmed previously identified associations with *IRF6*,* VAX1*,* NOG*, and *MAFB* (Sun et al. 2015). The first GWAS for cleft palate (CP) specifically identified a common missense mutation in *GRHL3* as significantly associated with CP (Leslie et al. 2016). Another study using a different but complementary targeted‐sequencing approach also associated the missense variant in *GRHL3* with CP (Mangold et al. 2016).

Evidence from OFC GWAS show that some loci are population specific and others are not (Beaty et al. 2010). The difference, mainly due to differences in allele frequency and in population specificity between ancestral populations, was demonstrated by the identification of risk loci at the chr8q.24 locus in Caucasians in Europe and North America and *MAFB* in Asians (Beaty et al. 2010). Targeted resequencing efforts around GWAS and known clefting genes identified a de novo damaging functional mutation in *PAX7* and noncoding functional mutations near *NOG* and *FGFR2* (Leslie et al. 2015). Given the successes of the cleft palate GWAS in identifying new risk loci and leveraging the smaller regions of linkage disequilibrium around susceptibility allele(s) in African populations, a GWAS of African populations of sufficient sample size could assist with the discovery of new risk loci, and data produced from this effort can be used to fine‐map risk loci identified in populations of European and Asian ancestry.

## Post‐GWAS Era

Exome sequencing, copy number variations, and whole‐genome sequencing studies are also now possible for studying complex traits like OFCs. Due to our ability to interpret the contribution of coding variants over noncoding variants, exome sequencing appears to be the most promising of these omics approaches. In 2014, Peyrard‐Janvid et al. conducted exome sequencing studies of a large family with VWS and identified *GRHL3* as a second candidate gene for VWS. This gene accounts for 5% of all VWS cases thus leaving 25% of VWS to be due to other genetics and genomics causes not yet identified. Exome sequencing of second‐ and third‐degree relatives of children with nonsyndromic OFC identified a shared rare damaging mutation in *CDH1* (Bureau et al. 2014). Additional rare mutations using a targeted sequencing approach have been identified in *CDH1*, suggesting that it is a nonsyndromic OFC candidate gene (Brito et al. 2015; Ittiwut et al. 2016). A recent cleft‐palate‐only exome‐sequencing study identified loss‐of‐function mutation in *ARHGAP29* and suggested a role for mutations in this gene in Mendelian forms of cleft palate (Liu et al. 2016).

Copy number variations have been reported to contribute to syndromic and nonsyndromic forms of clefts, as reviewed in Maarse et al. (2012). Moreover, a genome‐wide deletion‐association analysis identified a candidate locus near ch7p14.1 with a higher frequency in CL/P than in controls (Younkin et al. 2014). Analyses of genome‐wide inherited deletions identified a 67 kb deletion in *MGAM* on chr7q34 and a 200 kb spanning genes *ADAM3A* and *ADAM5A* on chr8p11 (Younkin et al. 2015).

## Epigenetics

Beyond variations in DNA, the epigenome is increasingly being reported as relevant to the etiology of diseases. This has been well established in cancer, obesity, and diabetes (Keil and Vezina 2015; Wahlqvist et al. 2015). The impacts of variations in DNA methylation and acetylation, otherwise known as epi‐mutations, have been reported to last beyond three generations (El Hajj et al. 2014). To date, despite evidence to show increased risk with active and passive maternal smoking (Little et al. 2004; Bille et al. 2007; Butali et al. 2013a,b), no genome‐wide DNA methylation study for OFC has been conducted. The mechanisms surrounding the impact of maternal smoking and of other environmental factors such as folic acids and alcohol are not clearly understood. It is possible that these factors are imprinted on the maternal genome and inherited by the developing embryo, leading to craniofacial malformations. For instance, folate has a huge impact on DNA methylation and activation of some genes that may be critical during a fetus's craniofacial development. Examining the methylation patterns in cleft individuals and families will be an additional approach to understanding the mechanism underlying etiology and development of OFCs.

## Conclusions

Using methods such as linkage, association studies, GWAS, exome sequencing, copy number variation and whole‐genome sequencing, we have taken several giant steps toward understanding the genetic causes of nonsyndromic clefts. Our understanding is critical for informing at‐risk families and providing specific counseling advice. It will also form the basis upon which we can explore the design of early diagnostic panels. With additional unbiased identification and understanding of the influence of environmental factors such as folic acid, we have the potential to modify risk in families while pursuing the ultimate humanitarian goal of preventing orofacial clefts.
